# Supplementary figures and images for: Understanding the epidemiology and pathogenesis of Mycobacterium tuberculosis with non-redundant pangenome of epidemic strains in China
Source: PLoS One. 2025 May 19;20(5):e0324152. doi: 10.1371/journal.pone.0324152 (PMC12143926; doi:10.1371/journal.pone.0324152)

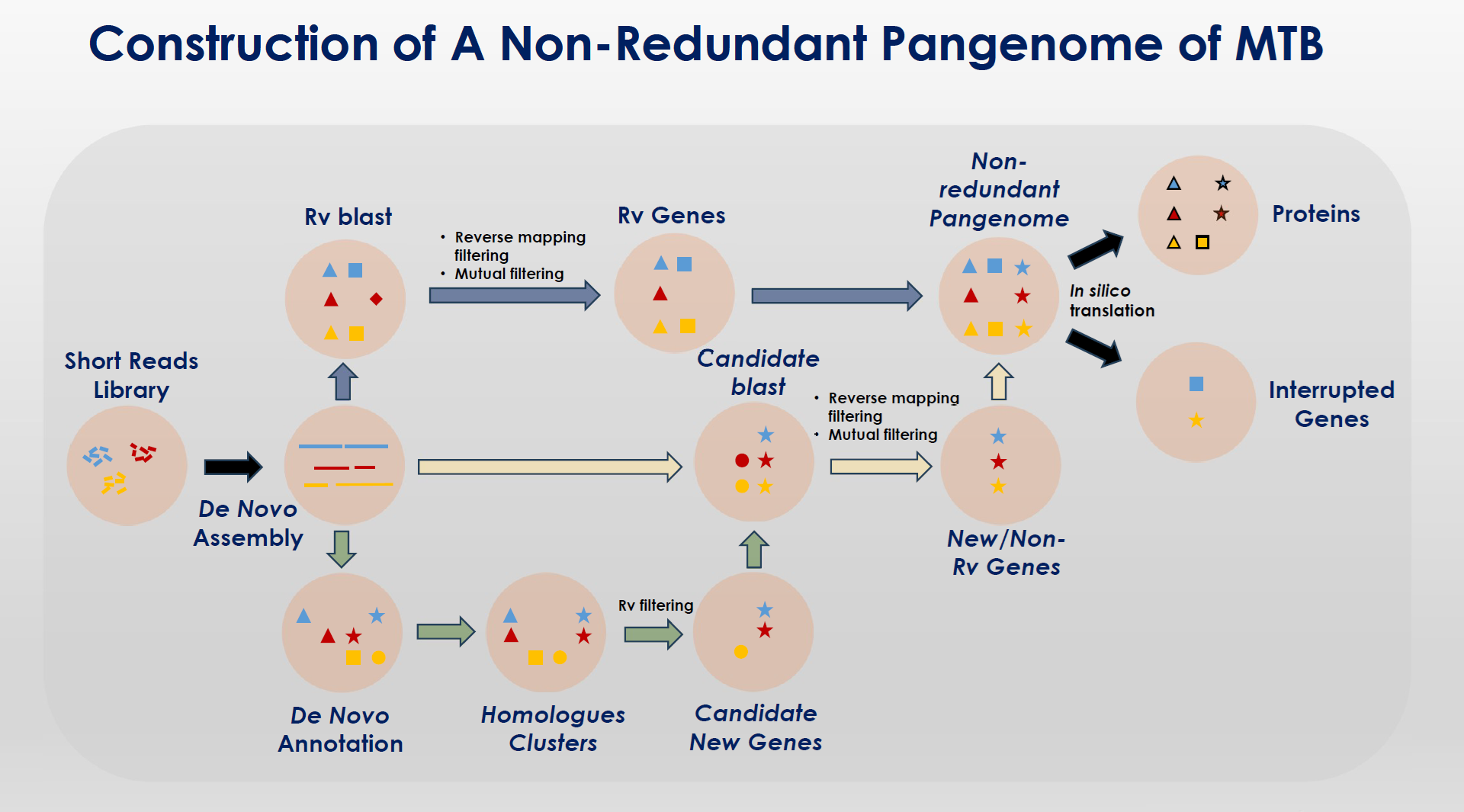

Supplement: S1 Fig — (TIF) [file pone.0324152.s001.tif]
