# Supplementary material for: Understanding the epidemiology and pathogenesis of Mycobacterium tuberculosis with non-redundant pangenome of epidemic strains in China
Source: PLoS One. 2025 May 19;20(5):e0324152. doi: 10.1371/journal.pone.0324152 (PMC12143926; doi:10.1371/journal.pone.0324152)
Supplement: Attachment — Submitted filename: response2reviewer.docx [file pone.0324152.s003.docx]

Please see the original reviewer’s comments and response (in grey shade and italic) below:

=========================================================================

PONE-D-24-47527
Understanding the epidemiology and pathogenesis of Mycobacterium tuberculosis with non-redundant pangenome and population genetics
PLOS ONE

Dear Dr. Zhou,

Thank you very much for submitting your manuscript to PLOS ONE, and for responding to our recent requests regarding your submission. After careful evaluation, we have decided that your submission does not meet our publication criteria and must be rejected.

Based on our evaluation of your manuscript and the information you provided, we do not feel that your submission meets our ethical requirements for human subjects research submissions. PLOS ONE requires that research meets all applicable standards for the ethics of experimentation and research integrity (http://journals.plos.org/plosone/s/human-subjects-research). We reserve the right to reject any submission that does not meet our internal ethical standards, which in some cases are more stringent than local ethical standards.

We note that your response to question 3 of the Clinical Studies Checklist states that you have reported the date(s) when you conducted this study. This is inconsistent with your manuscript, which after our most recent request does not specify the dates between which the study was conducted. It is therefore not clear whether you obtained the necessary ethical approval for the study to take place before data were accessed for this study. For human subjects research, PLOS ONE requires that authors obtain ethics committee approval before beginning the study, and we do not accept retrospective ethical approval.

***Response:***  *In this submission, we have added the information and modified the methods section accordingly (section Methods, Samping and WGS).*

As a result of these concerns, we cannot consider the manuscript for publication at PLOS ONE. I am sorry that we cannot be more positive on this occasion but hope that you understand the reasons for this decision.

Yours sincerely,

Athira Jayasree Kumar
Editorial Office
PLOS ONE
