# Supplementary material for: Understanding the epidemiology and pathogenesis of Mycobacterium tuberculosis with non-redundant pangenome of epidemic strains in China
Source: PLoS One. 2025 May 19;20(5):e0324152. doi: 10.1371/journal.pone.0324152 (PMC12143926; doi:10.1371/journal.pone.0324152)
Supplement: Attachment — Submitted filename: comments_JZ.docx [file pone.0324152.s004.docx]

Comparative genomics is arguably the best tool for querying the Mtb’s evolutionary trajectory and the functional consequences thereof. In this manuscript, the authors presented a comprehensive computational pipeline to infer Mtb’s pan-genome architecture using 420 public short-read sequencing data of Mtb strains isolated through a single national survey conducted in China. The pipeline appears reasonable based on the methods described, though the code used for the analyses was not provided. The analysis revealed a closed pangenome comprising 4,278 genes. The authors reported an interesting finding that among the 4,098 annotated protein-coding genes, only 1,651 encode full-length proteins across all genomes, while the remaining genes were disrupted in at least a subset of strains. Furthermore, the authors reported known and potentially new phylogenetically structured structural variations (SVs) and large structural variations (LSVs) and estimated gene-wise selective pressures using a single metric.

Overall, the manuscript is well-structured and could be a valuable asset to the TB research community. I’d be happy to read this manuscript again in its published format if the authors could address the following minor concerns:

1. Which H37Rv genome assembly was used as the reference? Several H37Rv assemblies now exist, including curated assemblies generated using long-read sequencing (e.g., DOI: 10.7554/eLife.97870.1). Since the analyses were based on a single annotated H37Rv genome, the authors should confirm that they used a complete, high-quality genome assembly with no blind spots. They should also provide the specific assembly ID of the H37Rv reference genome used in this study.

2. I commend the authors for the incredibly large amount of work they did to improve the overall power and robustness in calling SVs and other genetic variations by integrating a handful of existing bioinformatic tools. That being said, this is not the first Mtb pan-genome paper and there were several recent studies that used long-read sequencing to generate complete genome assemblies and conduct SV analysis, which were not cited in the present manuscript. Please cite these relevant studies, such as the recent eLife paper by Behruznia et al. (DOI: 10.7554/eLife.97870.1) and the preprint by Marin et al. (DOI: 10.1101/2024.03.21.586149). These studies demonstrated that incomplete contig assemblies from short-read sequencing can lead to biases in accessory gene calling. Comparing findings with these datasets could help avoid spurious variant calls, particularly those discussed in lines 295-297.

3. The number of coding genes with interruptions varies between different parts of the manuscript. For example, line 40 states 2,447 interrupted genes, while line 298 reports 2,194. It could be me who mis-interpreted the words but I do think that these numbers would benefit from explicitly stating the criteria used for counting interrupted genes.

4. Terms such as "unclassified interruptions" (lines 285-295) and "complex" (Table 1) are unclear. Additionally, frameshift mutations are referenced in lines 180-181 but seem omitted from Table 1. Please provide precise definitions for terms like "unclassified interruptions" and "complex" mutations, and, if applicable, expand the legend for Table 1 to explain each mutation category.

5. The rationale for using Tajima’s D to infer selective pressure is unclear, especially when extensive SVs are expected to skew the metric toward extreme values. Please justify why Tajima’s D was chosen over alternative methods that may be more robust to structural variation, such as the McDonald-Kreitman test or dN/dS ratios.

6. Lines 315-316 report 40 genes absent in lineage 2 strains, many of which are phage-related or CRISPR-associated. A recent study (DOI: 10.1128/spectrum.00527-24) identified an association between drug resistance and the absence of CRISPR genes. The authors may consider comparing the results to this published study and testing for convergence in findings.

7. The homoplastic SVs near the plcC locus are intriguing and have also been identified in the Marin et al. preprint. Please cite this relevant preprint and perhaps also discuss potential biological implications of homoplastic SVs in this genomic region.

8. Fig. 5 lacks a legend explaining the color notations, and Figure 4 was placed before Figure 1 in the manuscript. The authors should also consider including a supplementary data sheet corresponding to Figure 4, which would greatly benefit readers who want to perform an in-depth re-analysis of this information-rich dataset.
