# Supplementary material for: Understanding the epidemiology and pathogenesis of Mycobacterium tuberculosis with non-redundant pangenome of epidemic strains in China
Source: PLoS One. 2025 May 19;20(5):e0324152. doi: 10.1371/journal.pone.0324152 (PMC12143926; doi:10.1371/journal.pone.0324152)
Supplement: Attachment — Submitted filename: Response to Reviewers.docx [file pone.0324152.s005.docx]

**Responses to reviewer 1:**

**l.224 The pangenome is based on the population structure of Mtb strains in China. For instance lineage 3 (gene content) is completely missing. This limitation should be addressed in the title and the abstract.**

Thank you very much for your comment. We have made the modification accordingly in the title and abstract.

**l.40 and l 427 The statement that only like 50% of the core genome is intact and translated to functional proteins is difficult to follow from the results section. Usually, one allows some alleles to be missing or failing the thresholds in core genome schemes, so this number above seems just summing up disrupted ORFs in individual samples. If that is really an unusual finding, this needs to be presented better.**

We refer to core genes those present in all 420 strains + H37Rv (n=421). If some gene is missing in less than 5% of the sample population (400 <= n < 421), we call them soft-core genes. Based on this definition, there were 3,438 core genes including some core genes which might harbor SVs and other variations. If there is no SV or other high impact point mutations, such as frameshift mutations/non-sense mutations, detected in any strain for a core gene, we consider it as intact and translated in this pangenome. Thus, it’s not the sum of the disrupted ORFs, which is at the individual coding sequence level; the concept of 50% intact core genes is at strain level.

**l. 227 this paragraph is difficult to understand. Where is the number of 1 million predicted CDSs coming from when on average 4 thousand genes are predicted per genome? And what does it mean they were identical to the blast results? The whole selection procedure of genes included for the pangenome may benefit from a flowchart.**

The 1 million predicted CDSs is the sum of the number of annotated CDS in all strains. Each strain has been annotated by Prokka of about 4000 CDSs. There are 420 strains. Thus, in total about 1,680,000 CDSs.

By “identical to blast results”, we refer to the comparison between the annotated CDSs by Prokka and blastn results using Rv gene sequences as query sequences and assemblies as subject sequences. If these two results have identical sequences, we consider the annotated CDSs identical to blast results.

We have produced a flowchart (**Supplementary Fig 1.**) to illustrate the procedure of producing the pangenome and data analysis to make this approach clearer.

**l.285 I re-read this passage a couple of times, and could not grasp what the authors want to say here.**

In this paragraph we investigated the possible causes for the unclassified interruptions. Because 32.48% of the interruptions cannot be matched to detected variations, which is not a small proportion, we want to check whether it’s due to the flaw of our pipeline or other technical reasons.

After further looking into the data and literature, we found that about 71% of the unclassified interruptions are probably due to the characteristics of the raw data produced on Illumina platform for MTB genomes, which might be introduced by high GC content/repetitive sequences and the limitations of the platform; in the remaining unclassified interruptions (30% of the total), about 39% were concentrated in 33 (out of 420) strains, indicating these 33 strains have more un-classified interruptions than other strains which might be due to low quality in the sequencing data/assembly.

After excluding these two factors, limitations of sequencing platform and low-quality samples, only 8% (3,792/32,379) of the unclassified interruptions remain. This observation suggests that other sequencing platforms, such as PacBio or NanoPore, could improve the quality of pangenome dramatically.

**l.433 What is exactly the extend of structural variation as compared to SNPs in Mtb? You highlight RvD2 is that the known RD2 deletion in lineage 2? Any insides/observations on new (maybe phylogenetic) larger deletions?**

With the same sample set of 420 MTB strains, we have detected high confidence SNPs at 33,220 unique positions with reference to the H37Rv genome (NC_000962.3) using current standard pipeline (original citation 20). There are 76,671 SVs detected in 4098 Rv coding genes in the same sample set, corresponding to 7,334 unique SVs (besides IS*6110* insertions), but only 53,814 resulted in inactivation of coding genes in a subset of strains.

The RvD2 (length ~ 6,800 bp) is deleted in H37Rv due to IS*6110* insertions at position 1987702. RD2 (length = 10,787 bp) is deleted in BCG but presents in H37Rv genome from 2,221,064 to 2,221,064, which was not detected in this sample set. See original ref 7 for details.

We have discovered one recently reported LSP (4,494 bp, inserted to H37Rv at 2,219,418, line 306-308) and one new LSP (3,138 bp, inserted between 1,414,558-1,415,891 in H37Rv, line 303-306) first reported in this study which are lineage specific and with annotated CDSs.

**l.457 Also like 60% of your core genome is under (positive?) selective pressure? Mtb is so far thought to be shaped by purifying selection.**

Because we only calculated Tajima’s for Rv genes with high quality data (excluding 253 low quality genes), and DNAsp requires at least 4 sequences (with valid ORFs) to calculate Tajima’s D, only 3,742 coding genes obtained the value of Tajima’s D.

2,349 (62.77%) out of these 3,742 coding genes showed signal of selective pressures, thus about 60% of coding genes are under selective pressure, among which 878 were core genes. (Table 2). We measured positive selection by detecting homoplasy events. Among the 878 core genes under selective pressures, 292 genes have been detected of homoplasy SVs or SNPs indicating positive selection.

This conclusion is also supported by another study using dN/dS (original citation 66). This can be expected as MTB is in an environment facing constant challenges such as host immune attack and nutrient deficit.

Purifying selection certainly plays a big role in MTB’s evolution, but the impacts of purifying selection are not even among coding genes, as 540 out of 1,651 core genes (at protein level) are highly conserved (no variation or lower nucleotide diversity than average) in our sample set and 2,861 (~67%) genes have lower nucleotide diversity than the average level, while more than 1,000 genes show signal of positive selection as suggested by homoplasy.

**Minor comments:**

**l.63 the term “ancient” vaccine is maybe a bit degrading**

BCG vaccine was first used medically in 1921, only 25 years after the first vaccine developed in history and which is more than 100 years now, so we agree ancient is hardly justified we now refer to it as an “old” vaccine.

**l.65 pretomanid might be mentioned as well then with regard to new drugs**

Thank you for your suggestion. We have added this to the introduction.

**l.70 introduce MTB abbreviation at first mentioning**

Done.

**l.83 Bottai et al https://www.nature.com/articles/s41467-020-14508-5 demonstrated the increased virulence of strains with a deleted TbD1 region**

Thank you for your suggestions.

**l.106 I would not say that nonsense or frame shifts are ignored in presence/absence gene content analysis. Usually that involves a de novo annotation, and current algorithms still can link genes that are truncated or have frameshift mutations with their homologue/functional counterpart.**

We agree that the statement is confusing. We have changed this part to make it precise.

**l.123 explain selection criteria, what does it mean based on drug resistance patterns?**

We have added this information from lines 124 to 128.

**l.144 explain a bit why CDSs needed to be clustered, and what is meant with overlapping with Rv? That also affects the results part. How do you come to 10 thousand coding gene clusters?**

To find new genes not in the reference genome (H37Rv), each strain needs to be annotated to identify CDSs. However, annotation software will sometimes assign the same CDSs in different genomes to different names or choose alternative ORFs for the same CDSs. So, annotation for different genomes will not automatically match each other, but need be compared by their sequence in a pair-wise manner to find their matches in each genome. This step is called clustering. Algorithms developed for this process include COG and OMCL.

As mentioned above, sometimes alternative ORFs are chosen by annotation software; in other cases, interruptions or variations will result in fusing or splitting of ORFs. Some of these ‘new’ ORFs are transcribed/translated, but in most cases according to current understanding (though we don’t know to what degree for present), they are not. But clustering algorithms will treat them as completely new genes. This will result in an inflation of the number of accessory genes. That is why after clustering step, there are more than 10k CDSs in the pangenome. For this reason, we introduced the *blastn* step to remove this artifact.

*Blastn* step will find the best hit of each Rv gene in other genomes. According to this study (original citation 27), alternative ORFs overlapping > 60 bp with the “true” ORFs are mostly falsely called. So, if two ORFs annotated in different genomes found to be overlapping > 60 bp in one genome by *blastn*, one of them is discarded and the Rv gene was kept in this study.

**l.198 briefly explain the TajimaD and pi measurements, and homoplasy.**

We have added the description in the results section.

**l.214 what are “different” evolutionary patterns?**

Our data shows that purifying selection, positive selection and various selective pressures all play important roles in shaping the population structure and current landscape of genetic diversity of MTB. However, these factors demonstrate their impact at different levels in different genes.

Coding genes could be divided into five groups: 1) genes under selective pressures (n=2,349) including the 192 genes under strong selective pressures and the 35 genes showing high levels of homoplasy under strong selective pressures; 2) the highly conserved core genes (n=540) with no homoplasy or selective pressures detected; 3) genes showing no signal of selective pressures but with homoplasy detected (n=435); 4) genes only influenced by population stratification (n=202) showing neither signal of selective pressures nor positive selection, including lineage-specific genes; and 5) the other genes (n=319) in the pangenome which showed no signal of selective pressures, no homoplasy, and no population stratification. However, these four classes are not strictly mutually exclusive, as all these evolutionary forces impact every gene at different levels in different classes which resulted in the continuous and complex landscape of genetic diversity in MTB.

We have added this explanation to the results section “Over-represented gene categories following different evolutionary patterns” from line 348 to line 417 to make this point clearer.

**l.263 what is a valid ORF, and what is not valid?**

By “valid ORF”, we refer to sequences of coding genes that could be translated into protein sequences by *in silico* method such as Biopython. We have added this explanation to the method (line 180) to make it clear.

**l.266 what exactly do you mean with “how genetic variations affected the presence of absence at different levels in the pangenome”? do you mean by which genomic change certain genes were likely disrupted?**

By “different levels”, we mean at gene level and at protein level. Complete deletions of genes would remove this gene sequence from genomes, while other variations, such as frameshift mutations, would keep (part of) this gene sequence in the genome but result in no protein product. Thus, different genetic variations would result in different variations at different levels in the pangenome.

**l.270 I assume the 76k sequence variants are detected in the whole dataset from a reference mapping approach. That does not include SNPs here right? Because the sentence above you are mentioning SNPs as well? And then you mention again “high-impact” point mutations. I would suggest to be very strict and consistent with naming of mutations and describe from which analysis which numbers come from.1**

Yes. The 76k variations don’t include SNPs.

The high impact point mutations are in the SNP category other than SV.

We have modified the sentence from line 274 to 280 to make it clear.

**L.357 rpsL high nucleotide diversity as compared to what?**

The nucleotide diversity of *rpsL* was compared to the average (=1.51e-4) and *rplC* and *ddn*. *rplC* and *ddn* also show no signal of selective pressure and are related to drug resistance like *rpsL*, but these two genes show lower nucleotide diversity than *rpsL*.

**Fig3 what is allele frequence here? And segregating site count? Allele = gene position? Or allele = gene sequence? Segregating site = SNPs?**

Allele frequency refers to the frequency of different alleles in the sample population.

Allele = variant; segregating site = polymorphic positions.

**Fig4 and 5 are not readable in the online version (low resolution)**

We have uploaded separate files for figures in tiff format which should meet the requirements for online publication.

**Is Fig4 a phylogeny based on 127 shell genes? Which method? Seems to be a cladogram. In the caption it is stated it is based on 33k SNPs. In that regard one would rather use a ML or at least neighbor joining tree to have the genetic distances represented. Also the term shell gene is used for the first time here. Is that equivalent to the core genome here?**

The phylogeny tree is based on 33,220 SNPs in the whole reference genome which was published in our previous study (original citation 20). The tree was constructed using max likelihood method using MEGA. The genetic distance information was removed from the tree for better presentation of the data. We added this information to both the method and the legend of figure 4.

We have added the definition of core/soft-core/shell/cloud genes in the methods (line 187).

**Responses to reviewer 2:**

**1. Which H37Rv genome assembly was used as the reference? Several H37Rv assemblies now exist, including curated assemblies generated using long-read sequencing (e.g., DOI: 10.7554/eLife.97870.1). Since the analyses were based on a single annotated H37Rv genome, the authors should confirm that they used a complete, high-quality genome assembly with no blind spots. They should also provide the specific assembly ID of the H37Rv reference genome used in this study.**

We used the complete genome assembly of H37Rv of version NC_000962.3. We didn’t use the most recently published data in this study because NC_000962.3 is the most widely used reference genome of MTB so far and has been tested for its accuracy. Though updated version could be more precise, the differences are small and it has not been tested and widely accepted. Also, when this study started the mentioned updated genome sequences are not available yet. For reproducibility and rigorousness, we still used the most classic one. With the comparison between these two versions available, it would be possible to liftover the current coordinates to the updated version. We have added the version information in the methods.

**2. I commend the authors for the incredibly large amount of work they did to improve the overall power and robustness in calling SVs and other genetic variations by integrating a handful of existing bioinformatic tools. That being said, this is not the first Mtb pan-genome paper and there were several recent studies that used long-read sequencing to generate complete genome assemblies and conduct SV analysis, which were not cited in the present manuscript. Please cite these relevant studies, such as the recent eLife paper by Behruznia et al. (DOI: 10.7554/eLife.97870.1) and the preprint by Marin et al. (DOI: 10.1101/2024.03.21.586149). These studies demonstrated that incomplete contig assemblies from short-read sequencing can lead to biases in accessory gene calling. Comparing findings with these datasets could help avoid spurious variant calls, particularly those discussed in lines 295-297.**

Thank you for your comments. In our study we also found that de novo annotation from assembly generated from short reads sequencing would result in inflated number of accessory genes, which resulted from gaps between assemblies and alternative ORFs in annotation. Thus, we used blastn and filtering with overlapping > 60 bp criteria to remove this artifact (original citation 27 for details). After this step, the number of accessory genes decreased almost by two thirds. This is in line with the reported number in Marin’s study (gene gain decreased from 719 to 285).

In Behruznia’s study, they reached about the same number of pan/core genome size as our study (4,252 genes v.s. 4,278 genes, 3,639 strains v.s. 3,438 strains), though they used different dataset and pipelines. This is expected as MTB has a “conserved” genome and a clonal population structure. Especially, they applies several steps to optimize the results, such as collapsing diverse gene families, filtering contamination, merging fragmented gene segments and refinding missing genes with Panaroo and blastn, which are similar to our blastn and removing overlapping steps.

In line 295 – 297, we discussed the contribution of low-quality samples to the un-classified interruptions in coding genes, which only correspond to 3.6% (3,792/99,694) of the total interruptions identified. This bias will not make dramatic changes to the conclusions.

**3. The number of coding genes with interruptions varies between different parts of the manuscript. For example, line 40 states 2,447 interrupted genes, while line 298 reports 2,194. It could be me who mis-interpreted the words but I do think that these numbers would benefit from explicitly stating the criteria used for counting interrupted genes.**

The 2,447 interrupted genes with interruptions include 253 low quality genes. The 2,194 genes in line 298 didn’t include these low-quality genes, 2,447 – 253 = 2,194.

**4. Terms such as "unclassified interruptions" (lines 285-295) and "complex" (Table 1) are unclear. Additionally, frameshift mutations are referenced in lines 180-181 but seem omitted from Table 1. Please provide precise definitions for terms like "unclassified interruptions" and "complex" mutations, and, if applicable, expand the legend for Table 1 to explain each mutation category.**

By interruptions, we refer to any type of causes that result in failure of translation by *in silico* methods. If SVs or high impact SNPs are detected for the gene sequence, then this interruption is classified by the type of variation detected. If no variation is detected by our pipeline, then this interruption is un-classified.

By “complex”, we refer to two or more types of variation which are detected in one genome within the scope of one gene. For example, by “complex SV”, it may refer to a combination of “Indel” and “IS6110” detected; by “complex”, it refers to a combination of both SVs and high impact SNPs detected. We have added this information to the legend of table 1 and also the results.

Frameshift mutations are usually caused by small indels that disrupt the coding frames. We categorized this type of mutation to the group of “Indel”.

**5. The rationale for using Tajima’s D to infer selective pressure is unclear, especially when extensive SVs are expected to skew the metric toward extreme values. Please justify why Tajima’s D was chosen over alternative methods that may be more robust to structural variation, such as the McDonald-Kreitman test or dN/dS ratios.**

We calculated Tajima’s D only using valid ORFs, thus it’s not affected by interruptions such as SVs.

dN/dS measurement is used to detect positive selection in protein coding genes. However, dN/dS expects excessive nonsynonymous substitutions, while episodic positive selection may not elevate the nonsynonymous rate high enough for the signal to be detected. In addition, samples within the same species are also subjected to selective sweeps which is not covered by dN/dS analysis. In the case of selective sweeps, Tajima’s D will be significantly negative thus can be analyzed. (ref: “Beginner’s Guide on the Use of PAML to Detect Positive Selection | Molecular Biology and Evolution.” <https://academic.oup.com/mbe/article/40/4/msad041/7140562>.).

McDonald-Kreitman test also uses dN/dS ratios. Besides, McDonald-Kreitman test requires both within and between species data for analysis, while Tajima’s D can work on within species data only. We only have and analyze MTB data in our study.

**6. Lines 315-316 report 40 genes absent in lineage 2 strains, many of which are phage-related or CRISPR-associated. A recent study (DOI: 10.1128/spectrum.00527-24) identified an association between drug resistance and the absence of CRISPR genes. The authors may consider comparing the results to this published study and testing for convergence in findings.**

Thank you for your suggestions. I have added this part between lines 655-657.

**7. The homoplastic SVs near the plcC locus are intriguing and have also been identified in the Marin et al. preprint. Please cite this relevant preprint and perhaps also discuss potential biological implications of homoplastic SVs in this genomic region.**

Thank you for your suggestions. I have added the discussion between lines 675-677.

**8. Fig. 5 lacks a legend explaining the color notations, and Figure 4 was placed before Figure 1 in the manuscript. The authors should also consider including a supplementary data sheet corresponding to Figure 4, which would greatly benefit readers who want to perform an in-depth re-analysis of this information-rich dataset.**

Thank you for your suggestions. A supplement table is now added (**Supplement table 1**). A legend is added to Fig.5.
