# Supplementary material for: Understanding the epidemiology and pathogenesis of Mycobacterium tuberculosis with non-redundant pangenome of epidemic strains in China
Source: PLoS One. 2025 May 19;20(5):e0324152. doi: 10.1371/journal.pone.0324152 (PMC12143926; doi:10.1371/journal.pone.0324152)
Supplement: Attachment — Submitted filename: Response to Reviewers2.docx [file pone.0324152.s006.docx]

**Reviewer #1: Thanks for the clarification and improvements.**

**I found this statement in the abstract still a bit misleading:**

**"However, due to 99,694 interruptions in 2,447 coding genes, only 1,651 may be**
**translated in all samples, which dramatically reduces the number of active core genes."**

**(1) How many of those interruptions actually occur only in single isolates? Your core genome definition of 100% for >400 isolates is quite strict already. If you now count every isolate with a gene interruption, there are probably many singletons and possibly also artefacts included. When you use your softcore genome definition instead (or vice versa genes interrupted in >5% of all isolates), it would be more relevant and may hint to genes or sub-lineages which really make a phenotypic difference. Also we have no indication if those interruptions really abrogate the gene function (for this statement one should maybe consider only large InDels)**
**(2) In that regard we also saw that different tools miss or overinterpret structural variants. Visual inspection of a reference mapping helped to gain confidence for certain applications, and also BWA/GATK pipelines would identify at least small indels and confirm the results from the assembly used here.**

(1) 6,035 SVs only occur in single isolates in either coding genes or intergenic regions. In the 4,098 coding genes, 5,111 (69.69% of 7,334) only occur in single isolates. This level is comparable to the reported level of 47.5% only for small indels(1-40bp) in the study of Coll (Coll F, Preston M, Guerra-Assunção JA, Hill-Cawthorn G, Harris D, Perdigão J, et al. PolyTB: A genomic variation map for Mycobacterium tuberculosis. Tuberculosis. 2014;94(3):346–54.) We have added this information to the result section line 287.

Indeed, the reviewer is correct these 99,694 interruptions likely include many artifacts. These artifacts are assumed to be concentrated in the 253 genes prone to platform-wide bias as stated in the results. Following the reviewer's suggestion we have revised the text to make this clearer (line numbers 24-28 document with revisions): "However, due to 99,694 interruptions in 2,447 coding genes, we can only confidently confirm 1,651 of these genes are translated in all samples. Assuming a proportion of these interruptions are artifacts, the number of active core genes would still be much lower than 3,483."

(2) There is currently no pipeline which can detect SVs as efficiently as SNPs. However, this study (Kosugi S, Momozawa Y, Liu X, Terao C, Kubo M, Kamatani Y. Comprehensive evaluation of structural variation detection algorithms for whole genome sequencing. Genome Biology. 2019 Jun 3;20(1):117.) suggests that some tools show better performance than others and overlapping results from different algorithms will improve accuracy. We have chosen tools according to our experience and following this strategy, used both tools working with assembly (e.g., minimap) and alignment.

In addition, we found for small indels (< 70 bp), several tools have shown high consistency with each other (> 90%), especially for bcftools which has shown high confidence by cross-checking with other tools. For large deletions (e.g, > 1000 bp), when there is a discrepancy between SV tools and presence/absence matrix, we visualized the region with IgV to confirm it.
